# Supplementary material for: Phase-separating pyrenoid proteins form complexes in the dilute phase
Source: Commun Biol. 2023 Jan 7;6:19. doi: 10.1038/s42003-022-04373-x (PMC9825591; doi:10.1038/s42003-022-04373-x)
Supplement: Supplementary file 3 — Description of additional supplementary files [file 42003_2022_4373_MOESM3_ESM.pdf]

Description of Additional Supplementary Files

File name: Supplementary Data 1

Description: The source data behind the main figures in the paper.
